# MicroRNA expression in immune tissues of adult chickens after embryo stimulation with bioactive substances

**DOI:** 10.1038/s41598-023-30299-3

**Published:** 2023-02-22

**Authors:** Aleksandra Dunislawska, Elzbieta Pietrzak, Ramesha Wishna Kadawarage, Maria Siwek

**Affiliations:** Department of Animal Biotechnology and Genetics, Bydgoszcz University of Science and Technology, 85-796 Bydgoszcz, Poland

**Keywords:** Animal biotechnology, Animal physiology

## Abstract

The microbiota has a profound impact on the host organisms. The interaction between the host and its microbiota has an epigenetic mode of action. In poultry species, gastrointestinal microbiota might be stimulated before hatching. This stimulation with bioactive substances has a broad spectrum and long-term effects. This study aimed to examine the role of miRNA expression stimulated by host-microbiota interaction via administering a bioactive substance at the stage of embryonic development. This paper is a continuation of earlier research in the field of molecular analyzes in immune tissues after in ovo administration of bioactive substances. Eggs of Ross 308 broiler chicken and Polish native breed chicken (Green-legged Partridgelike) were incubated in the commercial hatchery. On day 12 of incubation, eggs were injected: the control group with saline (0.2 mM physiological saline), probiotic—*Lactococcus lactis* subsp. *cremoris*, prebiotic—galactooligosaccharides, and synbiotic—mentioned above prebiotic with probiotic. The birds were intended for rearing. miRNA expression analysis was performed using the miRCURY LNA miRNA PCR Assay in the spleen and tonsils of adult chickens. Six miRNAs differed significantly, at least between one pair of treatment groups. The most miRNA changes were observed in the cecal tonsils of Green-legged Partridgelike chickens. At the same time, only miR-1598 and miR-1652 showed significant differences between the treatment groups in the cecal tonsils and spleen of Ross broiler chickens. Only two miRNAs showed significant GeneOntology (GO)enrichment with the ClueGo plug-in. gga-miR-1652 target genes showed only 2 GOs significantly enriched: chondrocyte differentiation and early endosome. gga-miR-1612 target genes, the most significant GO was regulating the RNA metabolic process. The enriched functions were associated with gene expression or protein regulation, the nervous system, and the immune system. Results suggest that early microbiome stimulation in chicken might regulate the miRNA expression in different immune tissues in a genotype-dependent manner.

## Introduction

Host microbiota interaction is a well-known and accepted phenomenon. Commensal microorganisms have a significant impact on host organisms^1,2^. Young organisms receive microbiota from their parents. However, in modern chicken production, such an interaction between parents and their offspring is disturbed. Fertilized eggs are incubated in commercial hatcheries in clean conditions^3^. The egg-hatching technology interrupts young chickens' natural way of acquiring commensal microbiota. At the same time, the specificity of chicken embryo development outside the mother organism allows an early modulation of gut microbiota before the hatching. The host-microbiota composition might be modulated with various bioactive substances, e.g., prebiotics, probiotics, synbiotics, and postbiotics^4^. The bioactive substances are introduced into the developing embryo using in ovo technology. Such stimulation of host gut microbiota at day 12 of chicken broiler embryo development proved to have long-term and a broad spectrum of effects on growth traits, feed efficiency, intestinal morphology, meat microstructure and quality, immune system development, physiological characteristics, the transcriptome and proteome^5–8^.

The impact of in ovo-delivered synbiotics on day 12 of egg incubation on gut microbiota composition and transcriptome of spleen and cecal tonsils throughout the rearing period was confirmed by microbiota population analysis and gene expression patterns in Cobb broiler chickens^9^. Changes in the transcriptome of the spleen and cecal after prebiotic (inulin) and synbiotic (inulin with *Lactobacillus lactis* subsp. *cremoris*) delivered in ovo on the day 12 of embryo development were detected starting from day one post-hatch until day 35 post-hatch^7^. Moreover, analysis of gene expression in the intestinal mucosa related to tight junctions and immune response demonstrated downregulation as an indirect effect of microbiota stimulation with galactooligosaccharide prebiotic administered in ovo in Ross broiler chicken was also proved by Slawinska et al.^10^.

Host microbiota interaction presents an epigenetic mode of action. Host organisms respond to the signals of microorganisms, in particular bacterial short-chain fatty acids. Epigenomic modifications allow for changes in host gene expressions without modifying the genetic code^11^. This mode of action in mammalian species is well described in the literature^11–13^. An epigenetic mode of action upon early microbiome modification in poultry has also been suggested^14^. Several mechanisms are related to the epigenetic regulation of gene expression, such as DNA methylation, histone modification, and miRNA^15^. Our previous study proved negative regulation of metabolic gene expression (*ANGPTL4*, *NR4A3*, *CYR61*) upon an early stimulation of host gastrointestinal microbiota with *Lactobacillus*-based synbiotics is related to DNA methylation. These mechanisms of epigenetic regulation of gene expression mediated by in ovo injection on day 12 of egg incubation were detected in the chicken liver^16^. The second possible mechanism of epigenetic regulation is miRNA. These molecules are small noncoding RNAs. Their length is from 18 to 25 nucleotides. They affect the protein level of the target mRNA, leaving the gene sequence untouched^17^. The impact of host microbiota stimulated with bioactives on miRNA profiling in the chicken liver was defined by Sikorska et al.^18^. It has been shown that probiotic and synbiotic administered on day 12 of egg incubation has a significant impact on miRNA expression activity in the liver of adult chicken^18^.

The mechanism of DNA methylation related to the regulation of gene expression under the influence of host-microbiota interaction was detected in the spleens of two different chicken genotypes^19^. That study proved epigenetic regulation depends on the bioactive substance modulating the host microbiota and the host genotype. Therefore, the goal of the presented study was to verify if the miRNA is responsible for the epigenetic gene regulation in the spleen and cecal tonsils originating from two different chicken genotypes stimulated in ovo with bioactive substances.

## Results

### miRNA expression analysis

**Figure 1:**
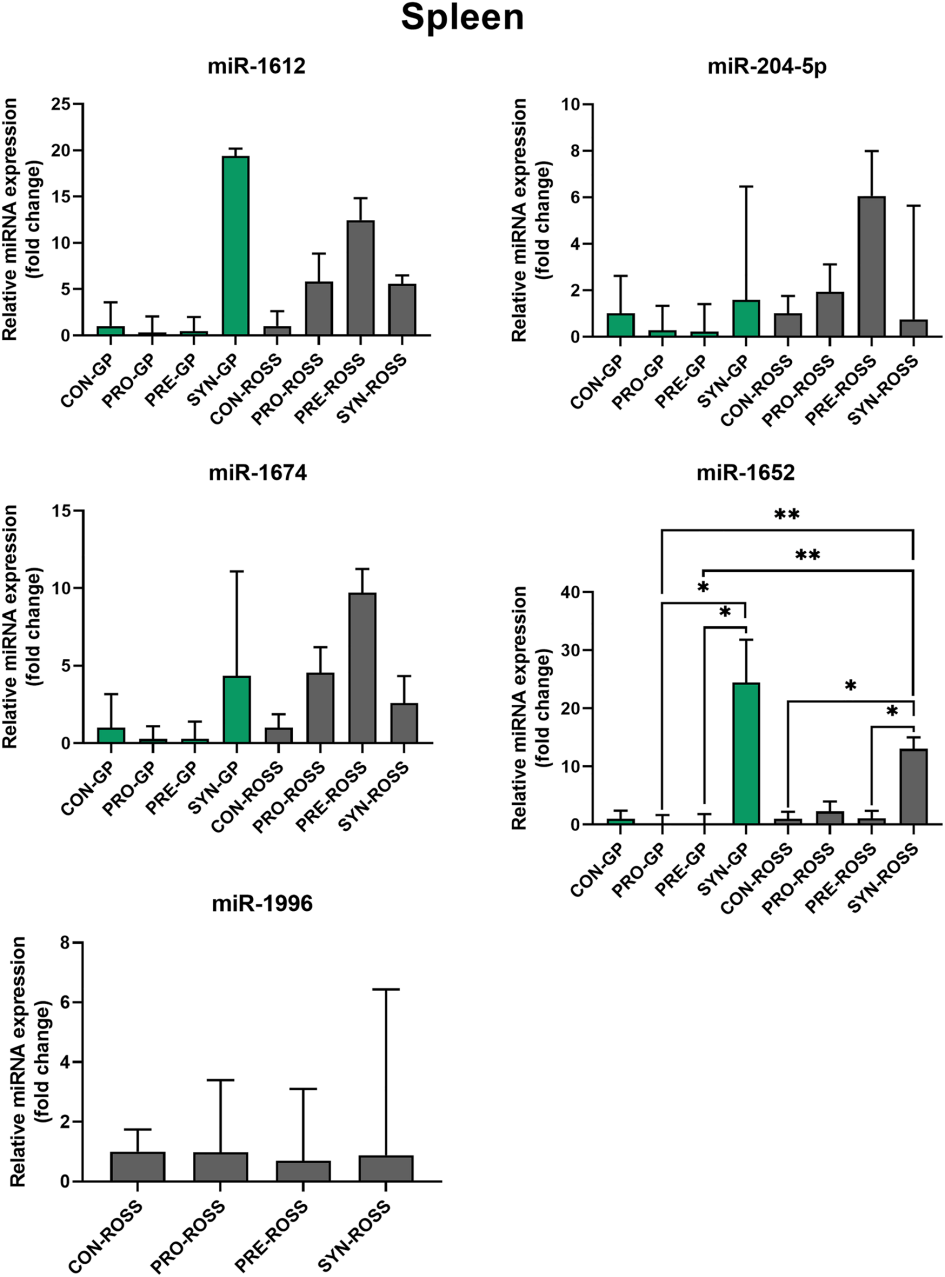
The Relative microRNA (miR) expression in the spleen of Green-legged partridgelike chickens (GP) and ROSS 308 broilers (ROSS) injected in ovo with saline-CON, probiotic-PRO, prebiotic-PRE, and synbiotic-SYN.

The ddCt values from qPCR analysis revealed the expression of six miRNAs (miR-1612, miR-1652, miR-1674, miR-1598, miR-1996, miR-204-5p) was significantly different at least between one pair of treatment groups according to the ANOVA analysis. However, most miRNA changes were observed in the cecal tonsils of GP chickens, while only two miRs showed significant differences between the treatment groups in the cecal tonsils and spleen of Ross chickens, respectively. A numerical increase in the expression of miR-1612 and miR-1674 was observed in the GP spleen after the administration of the synbiotic and in Ross after the administration of the prebiotic. Interestingly, GP expression decreased relative to controls after probiotic and prebiotic administration. A significant increase in miR-204-5p expression in the spleen was shown after prebiotic administration in Ross, while expression decreased after probiotic and prebiotic administration in GP. MiR-1652 expression was numerically and statistically significantly increased in the spleen after administration of the synbiotic in both genotypes. There was also a significant decrease in expression after prebiotic and probiotic administration in GPs. MiR-1996 expression was detected only in Ross, while these changes did not differ numerically from controls either. In the cecal tonsils, a numerical increase in the expression of all analyzed miRNAs was observed after administration of all substances to GP, while in Ross the numerical increase of all miRs occurs after administration of the probiotic. The fold changes of these six miRNAs in treatment groups, compared to the control group are illustrated in Figs. 1 (spleen) and 2 (cecal tonsils).

**Figure 2:**
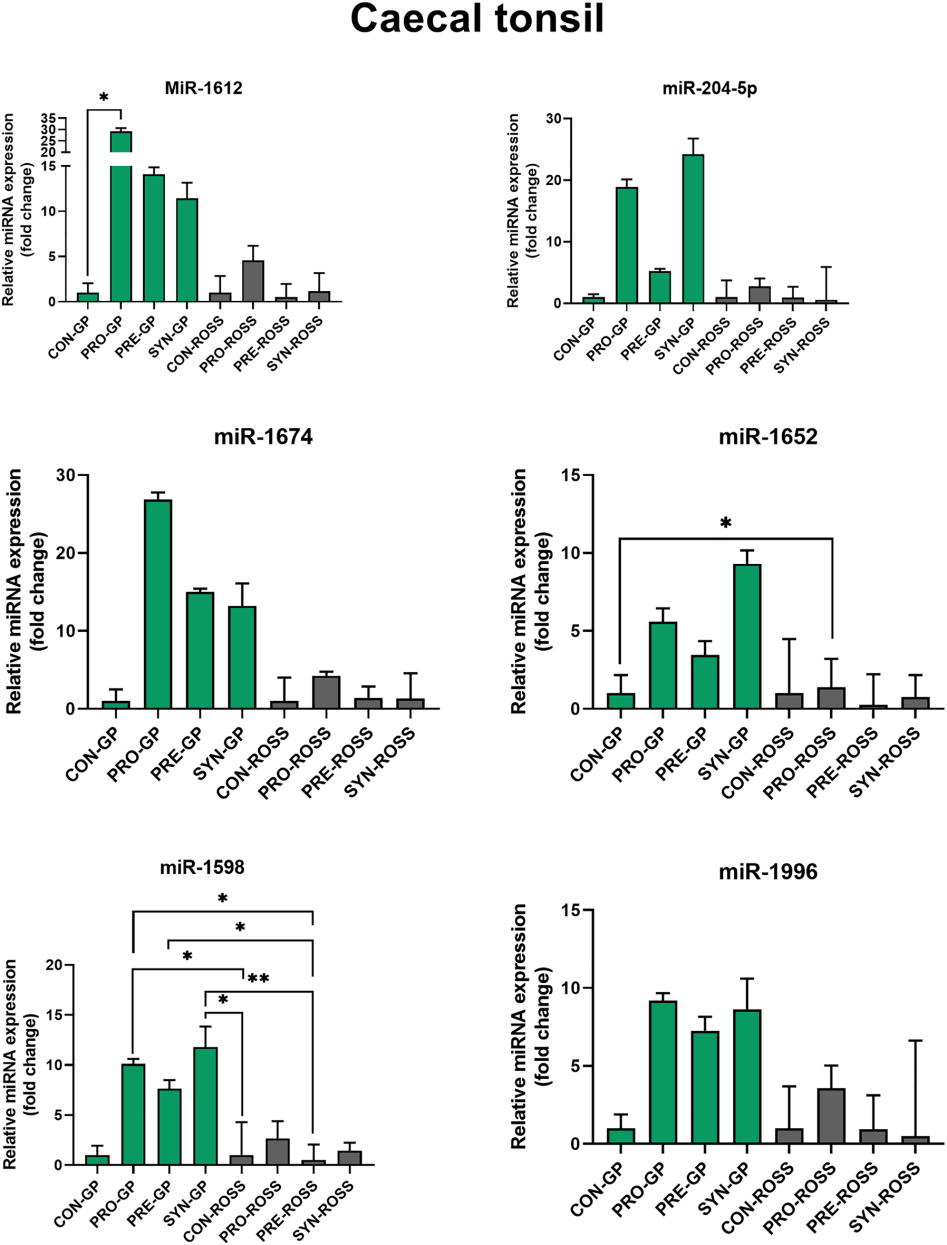
The Relative microRNA (miR) expression in the caecal tonsil of Green-legged partridgelike chickens (GP) and ROSS 308 broilers (ROSS) injected in ovo with saline-CON, probiotic-PRO, prebiotic-PRE, and synbiotic-SYN.

### Functional analysis

**Figure 3:**
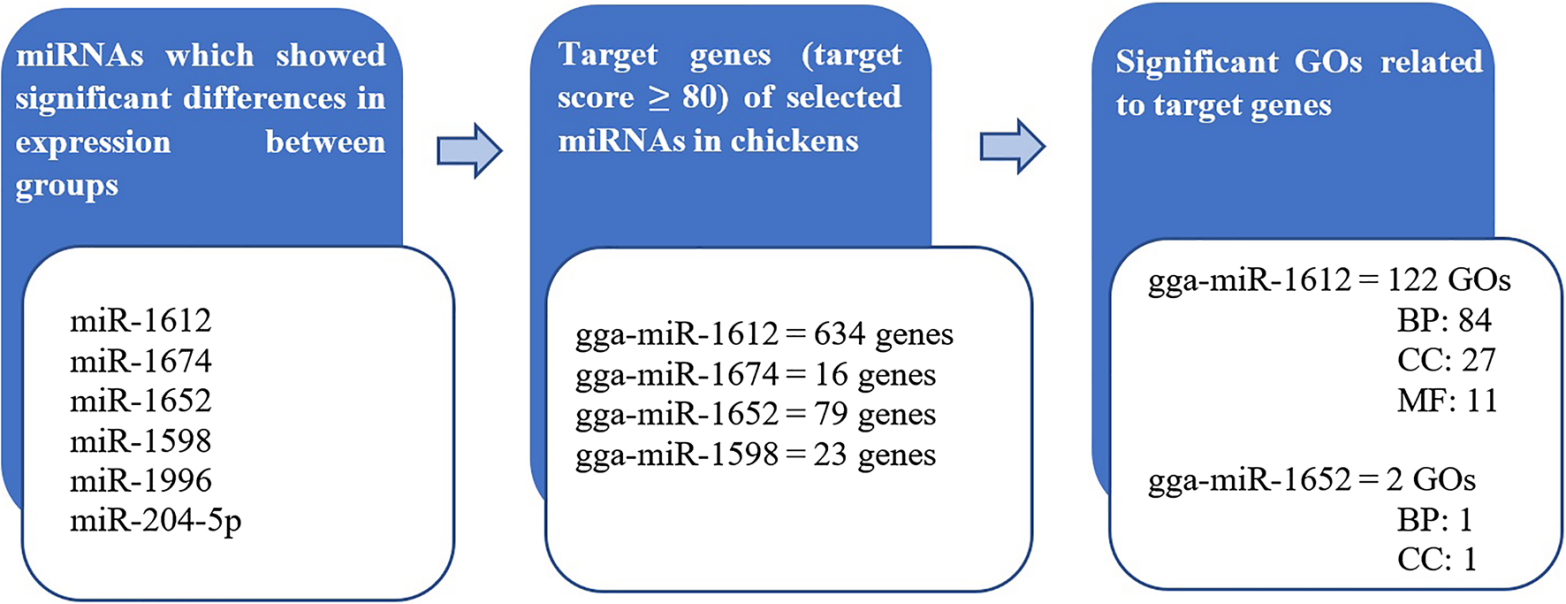
The summary of results from the functional analysis workflow.

Out of six miRNAs that showed significantly different results between treatment groups, the target genes of only four miRNAs were found in the miRDB database. However, only two miRNAs showed significant GO enrichment with ClueGo plug-in. The summary of the results along the workflow of functional analysis is shown in Fig. 3.

**Figure 4:**
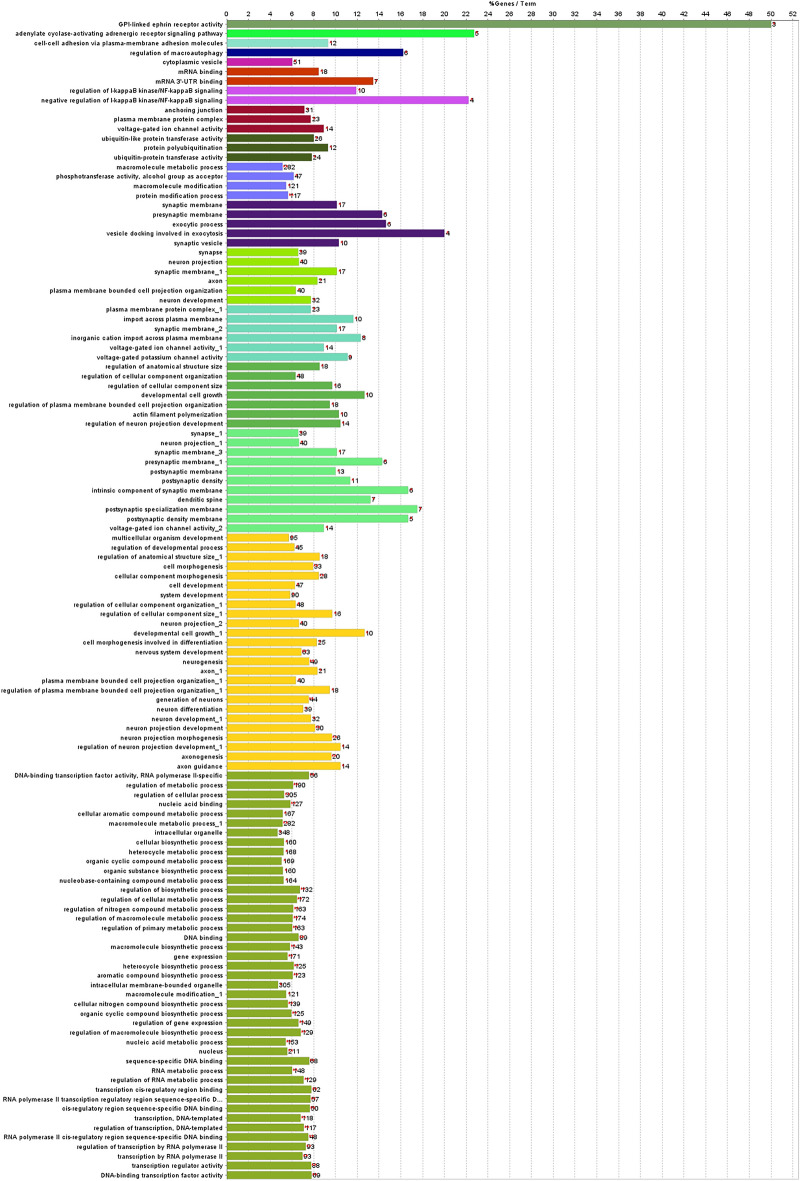
Results for enrichment analysis for target genes of gga-miR-1612: percentage of genes per significant GO.

**Figure 5:**
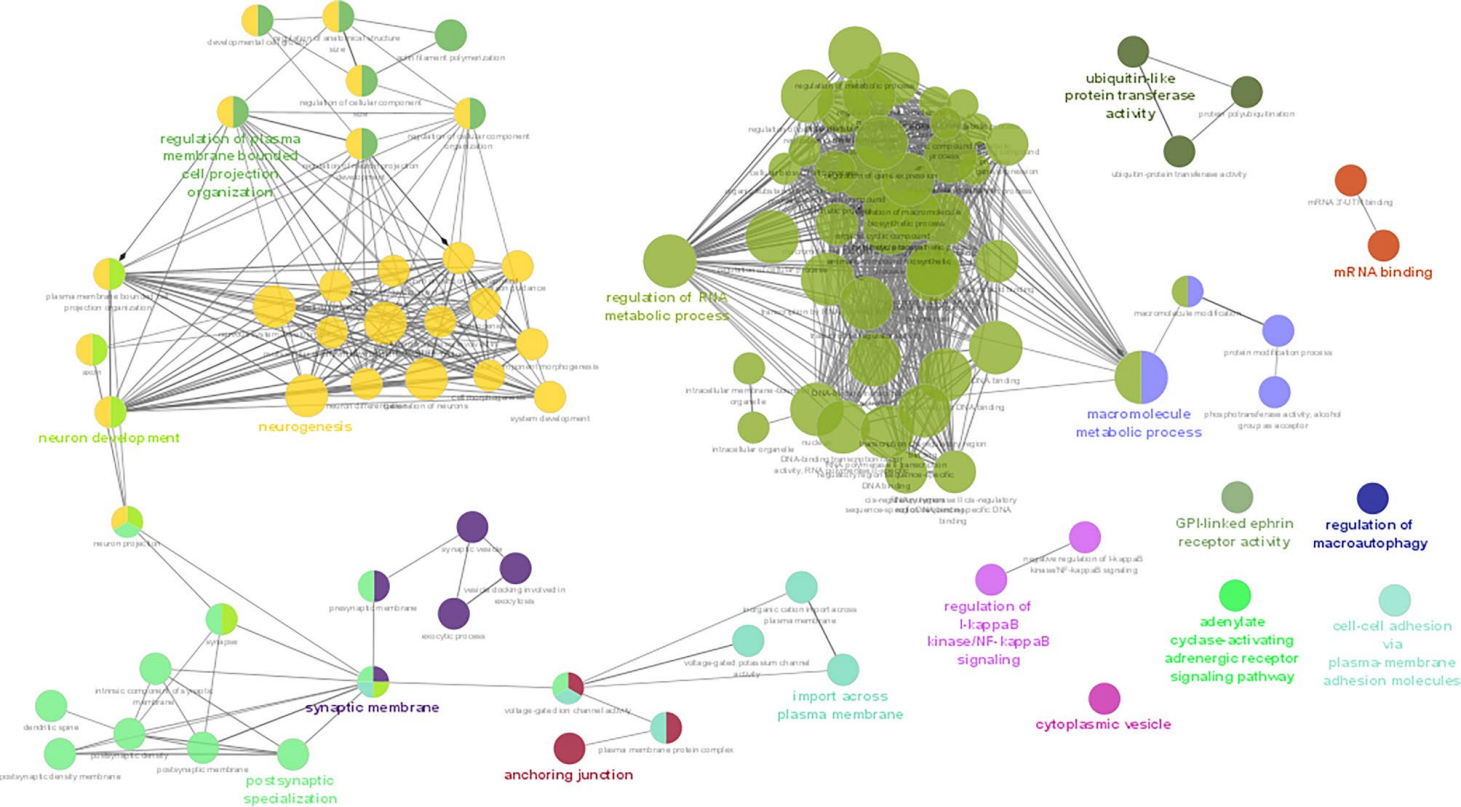
Results for enrichment analysis for target genes of gga-miR-1612: GO networks and Kappa score groups of GOs.

**Figure 6:**
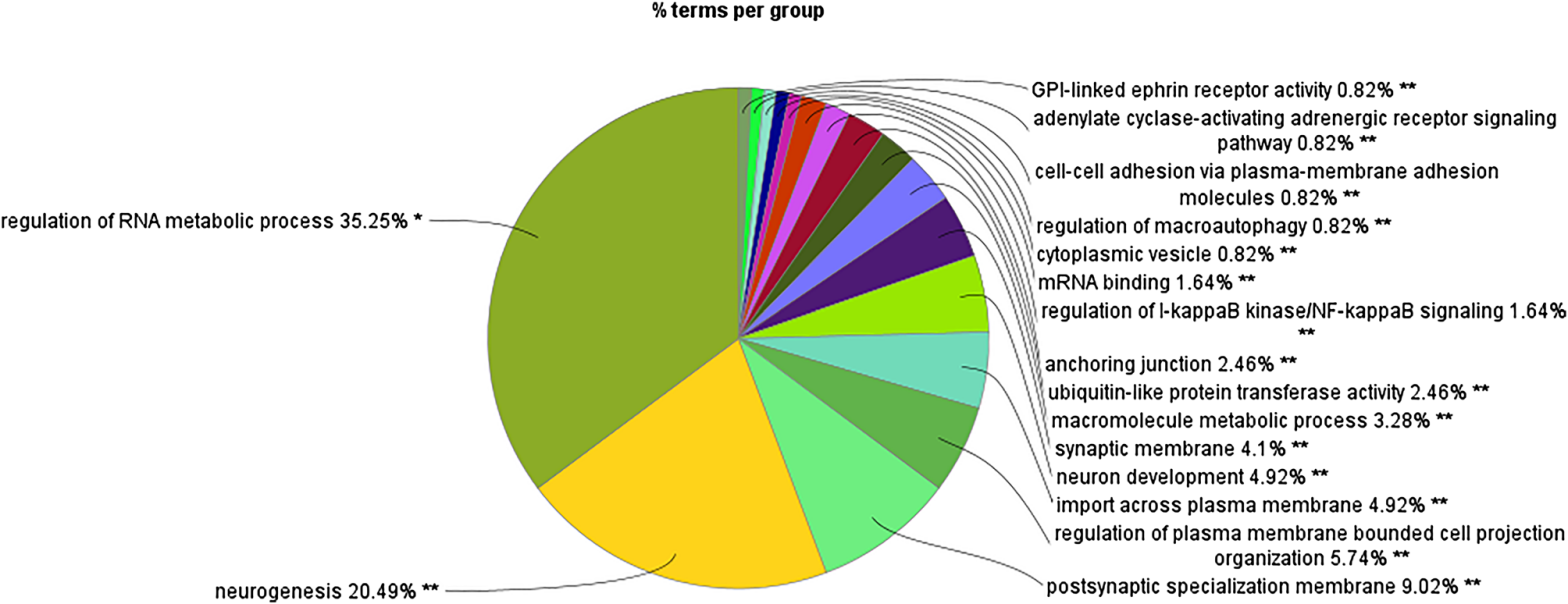
Results for enrichment analysis for target genes of gga-miR-1612: percentage of GOs for Kappa score groups.

**Figure 7:**
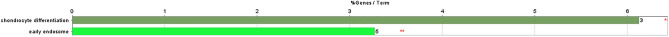
Results for enrichment analysis for target genes of gga-miR-1652: percentage of genes per significant GO.

**Figure 8:**
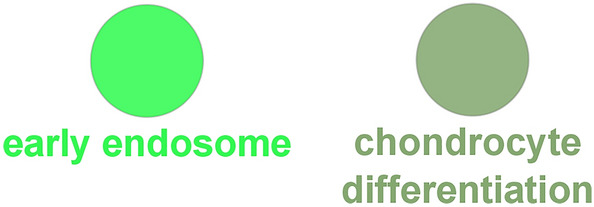
Results for enrichment analysis for target genes of gga-miR-1652: GO networks and Kappa score groups of GOs.

**Figure 9:**
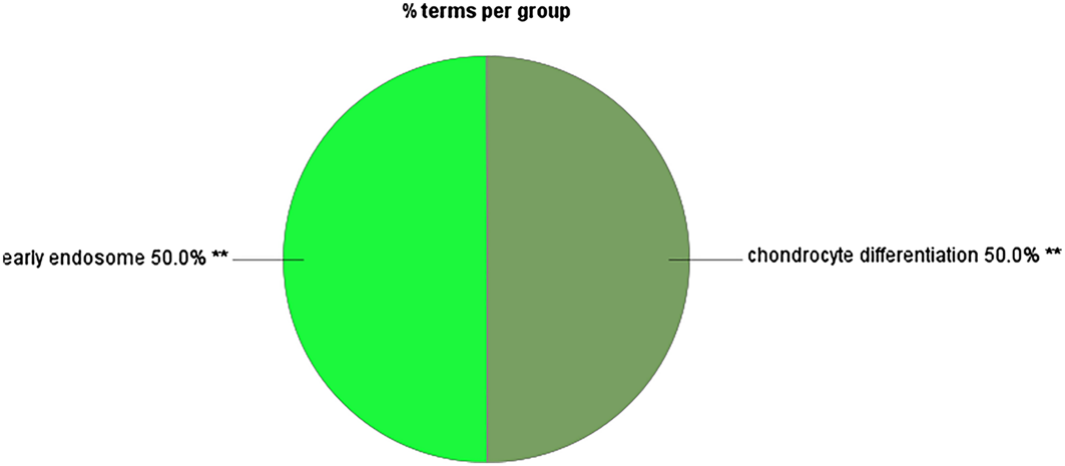
Results for enrichment analysis for target genes of gga-miR-1652: percentage of GOs for Kappa score groups.

The enrichment analysis showed 17 and 2 kappa score groups of enriched GOs for target genes of gga-miRNA-1612 and gga-miRNA-1652, respectively. The enrichment analysis results for gga-miR-1612 and gga-miRNA-1652 are shown in Figs. 4, 5, 6, 7, 8 and 9, respectively.

As per the enrichment analysis results of gga-miR-1612 target genes, the most significant GO was the regulation of RNA metabolic process, which showed the highest number of related GOs and highly significant within the Kappa score group. The Kappa score groupings showed that the functions associated with gene expression or protein regulation, nervous system, and the immune system were enriched. However, gga-miR-1652 target genes showed only 2 GOs significantly enhanced, namely chondrocyte differentiation and early endosome.

## Discussion

The current study is the follow-up analysis of the epigenetic mechanism related to gene expression in immune-related tissues of chickens modulated in ovo with bioactive substances^19^. According to our knowledge, this is the first comprehensive analysis of epigenetic regulation of bioactives administered in ovo on immune-related tissues (spleen, cecal tonsils (CT)) in two different chicken genotypes. Stimulation was performed on day 12 of egg incubation. As our numerous studies prove, this time is optimal for in ovo stimulation with bioactive substances because, during this time, the embryo undergoes intensive development^5^. It has been shown that in ovo stimulation on the day 12 of egg incubation with prebiotics, probiotics and synbiotics affects phenotypic traits in poultry^20^, development of immune organs (bursa of Fabricius and spleen), as well as increased proliferation of lymphocytes in the thymus^21^, influences the composition of the intestinal microbiota and morphological parameters of intestines in adult chickens^8,22,23^. The effect of in ovo stimulation on day 12 of egg incubation on the kinetics of gene transcripts related to the immune response, stress response (heat stress), and metabolism^23–26^. Recently, we have also focused on assessing the impact of in ovo stimulation on the day 12 on mechanisms related to the regulation of gene expression that may be subject to epigenetic inheritance. We have confirmed this effect on DNA methylation in spleen tissue^19^ and miRNA activity in the liver^18^. These results allowed us to hypothesize about the potential impact of early in ovo stimulation on the kinetics of expression of selected miRNAs in lymphoid tissues, both in the line of fast-growing chickens and the Polish native breed, which was empirically confirmed in this experiment. A previous study reported changes in the global and gene methylation level after administering prebiotics, probiotics, and synbiotics in ovo^19^. It was observed that genes were downregulated in the spleen and potential methylation-mediated regulation occurred when synbiotic stimulation was performed in ovo. There are also several reasons to link miRNA activity with DNA methylation by interacting with newly formed mRNA strands of a target gene. The literature shows that miRNAs regulate the major methyltransferases in animals. DNTM13B gene expression in chickens was post-transcriptionally regulated by five miRNAs^27^. In our previous study, methylation changes were also associated with the negative regulation of expression of genes related to immune response after prebiotic and synbiotic administration in broiler chickens (*SYK*, *ANGPTL4*, *IKZF1*)^19^. Methylated genes regulate several biological processes including innate and adaptive immunity, specification and the maturation of the T-lymphocyte. In the current experiment, it was observed that in ovo stimulation with bioactives will result in more modifications at the miRNA expression level in GP chickens compared to Ross broilers. Therefore, it can be suggested that the epigenetic effects of in ovo bioactive treatments depend on the chickens' genotype. A similar pattern was observed in relation to DNA methylation for gene expression in the spleen in these two different genotypes upon the modulation of host microbiota with bioactive substances in our previous study^19^. This is also in compliance with the previous study where GP and Ross chickens showed differential modifications to immune cells in the spleen and cecal tonsils^28^. Differences in responses by epigenetic mechanisms may result from the birds' genetic background and environmental tolerance. GP is a dual-purpose chicken breed, whereas GP belongs to the Polish native chicken breed under the genetics resources conservation program and it is known for its high disease resistance and adaptation to harsh environments. These breeds are maintained with no selection scheme^29^. Ross 308 is a meat-type chicken characterized by excellent production parameters. As all modern broiler chickens, also Ross 308 is a product of an intensive selection program. There is more likely another difference between these two chicken genotypes. According to study of Koenen et al.^30^ broiler and layer differ in their immune responses. Broilers such as Ross 308 mount strong short term humoral responses. GP which rather resembles the layer type of a chicken mounts long-term humoral responses in combination with cellular responses. Due to the possibility of regulating gene expression at the post-transcriptional level, miRNAs ultimately affect the number of individual proteins in the organism. The high activity of miRNAs associated with the immune system or the suppression of the expression of immune-related genes like a cytokines may prove to be extremely important for poultry production. Keeping the immune system activity can be energetically costly, resulting in stunted growth^31^. As shown during the selection of miRNAs for testing, the selected miRNAs are associated with regulating a large number of genes (from 271 to 1876 genes). The functional analysis showed that the selected microorganisms are related to metabolic and immunological functions and the nervous system. However, functional links in this area require further research.

Considering two immune-related tissues, CT and spleen, most of the modifications to miRNA expression were observed in cecal tonsils. At the same time, synbiotic was the treatment that caused most of these modifications in GP chickens. Accordingly, the current study suggests that the CT in GP chickens are more likely to undergo epigenetic changes when stimulated with synbiotics in ovo. The literature proves that the probiotic can participate in the interaction between the microbiota and the host and affect the expression of miRNAs^32^. Brisbin et al.^33^ showed that different probiotic strains display different potentials to induce immune effects in spleen and CT cells in vitro. Additionally, miRNA, an essential regulator of host gene expression, can be significantly associated with a given microbial community and even specific groups of bacteria. As evidenced in the literature, host miRNAs can enter a bacterial cell to promote or inhibit the growth of specific bacteria^34,35^. Studies on mice have also observed an increase in the expression of miRNAs responsible for alleviating inflammation after prior administration of *Lactobacillus plantarum* probiotic^36^. Therefore, it would be interesting to study the potential of different strains of probiotics in inducing epigenetic modifications at miRNA levels in other gut-related immune tissues to optimize the gut health of chickens via microbiome modulation.

In the current study, the expression of both miR-1612 and miR-1652 was increased in the CT of GP chickens. As the translation of the target genes are usually repressed by miRNAs by binding to the 3’-UTR (untranslated region) of the mRNA, in this case, it can be assumed that the increment of the expression of these miRNAs negatively regulates the GO enrichments associated with the target genes of these miRNAs. However, this issue requires further analysis of the regulation of gene expression by given miRNAs. The target genes for miR-1652 enriched two GOs: early endosomes and chondrocyte differentiation. Though chondrocyte differentiation does not make sense in the CT, the genes included in this GO showed us their potential role in immune responses. Based on functional analysis, it can be speculated that increased expression of miRNA can indirectly link to the downregulation of signaling pathways in CT when GP chickens are stimulated with synbiotics in ovo. However, the enrichment of early endosomes has a direct role in the immune system's functioning^37^. Therefore, with an increase in miR-1652, our results indicate a potential epigenetic downregulation of the immune functions in the CT in GP chickens upon in ovo stimulation with synbiotic.

MiRNA-1612 showed many target genes, thus enriching the more significant number of GOs. However, the functional kappa groups enriched could be categorized broadly into nervous and immune system functions while it employed many significant GOs related to gene expression regulation. The expression of miR-1612 was increased significantly in the CT of synbiotic-treated GP chickens but not Ross broilers. Hence, it appears that the synbiotic stimulation might have downregulated the energy costing nervous and immune excitements in CT, posing a less energy burden in GP chickens. A similar pattern was observed in a previous study which elucidated the methylation of immune related genes in the spleen of the same chicken breeds treated with the same bioactive substances^19^. It was observed that immune-related genes (*SYK*, *TNFRSF14*, *IKZF1*, *NR4A3*) were downregulated in the spleen, along with potential methylation mediated regulation when synbiotics stimulation was performed in ovo. There, the effects were more pronounced in Ross broilers than GP chickens; hence, we claimed that GP chickens are more environmentally adapted and can be less sensitive to microbiome stimulation. However, when looking at the results at the miRNA level in CT, the current study contradicts this claim where GP chickens showed a higher sensitivity to synbiotic stimulation with many significant modifications. Nonetheless, the miRNA modifications of the spleen were less pronounced in both chicken breeds.

The study's results suggest that microbiome stimulation might regulate the functions of the gut via different epigenetic mechanisms in different immune tissues in a genotype-dependent manner. Hence, bioactive substances and genotype should be considered for optimizing the benefits of microbiome modulation in chickens. The results presented in this publication are the basis for the analysis of miRNA regulation mechanisms and their target genes after the administration of bioactive substances in ovo on day 12 of egg incubation in broiler chickens.

## Methods

### Experimental design

A detailed experimental setup was described by Dunislawska et al.^19^. The collected tissues came from the experiment described in the publication^19^. This study is a continuation of the analysis. Briefly, 600 eggs of Ross 308 broiler chicken and 600 eggs of Polish native breed chicken [Green-legged Partridgelike (GP)] were incubated in the commercial hatchery. On day 12 of incubation, eggs were distributed into four groups: (1) the control group injected with saline (0.2 mM physiological saline) (CON), (2) probiotic—*Lactococcus lactis* subsp. *cremoris* IBB477 (PRO), (3) prebiotic—galactooligosaccharides (GOS; Bi2tos; Clasado Biosciences, Ltd., Reading, UK) (PRE), and (4) synbiotic—mentioned above *Lactococcus lactis* subsp. *cremoris* with GOS (SYN). Each egg was injected into an air chamber with 0.2 mL of an aqueous solution of the suitable substance. After hatching, birds were kept at the experimental station of the University of Life Sciences in Wroclaw, Poland.

### Tissue collection and RNA isolation

Five randomly selected individuals from each group were sacrificed by decapitation (cut between the first cervical vertebra and the occipital condyle) on day 42 post-hatching. Before slaughter, the birds were stunned (concussion/blow to the head) following Dz. U. I. 303 of 18 November 2009 (Annex IV. Methods of killing animals). Post-mortem tissues were collected for analysis, including the cecal tonsils and spleen. Tissues were stored in 5 mL Eppendorf tubes with stabilization buffer (fix RNA, EURx, Gdansk, Poland) according to the manufacturer's protocol. The experiment was approved by the Local Ethics Committee for Animal Experiments (Bydgoszcz University of Science and Technology, Poland) (study approval reference number 16/2014). All methods were carried out following relevant guidelines and regulations.

### miRNA profiling

RNA isolation was prepared using TRI reagent (MRC, OH, USA) and a commercial kit for additional RNA purification (Universal RNA Purification Kit, EURx, Gdansk, Poland) according to the manufacturer's protocol. Tissues were homogenized with the TissueRuptor II homogenizer (Qiagen GmbH, Hilden, Germany) in TRI reagent. RNA was validated quantitatively and qualitatively. The isolated RNA was analyzed by the LNA method. miRNA panel was selected based on two sets of microarray data from previous experiments (Chicken Gene 1.1 ST Array Strip, Affymetrix, Santa Clara, CA, USA) for spleen and cecal tonsils and was described by Sikorska et al.^18^. These data sets contained broiler chicken transcripts generated from individuals which received prebiotics and synbiotics in ovo on day 12 of egg incubation^10,24^. The selection of miRNAs for analysis was made by means of quantitative analysis of genes (based on the TargetScan software), which are regulated by the given miRNAs. Subsequent analyses were performed with the miRNAs regulating the highest number of genes. Results of miRNA expression after in ovo stimulation read from raw microarray data and quantification are presented in Table 1. miRNA expression analysis was performed using the miRCURY LNA miRNA PCR Assay (Qiagen, Hilden, Germany) according to the manufacturer’s protocol using miRCURY LNA miRNA SYBR Green qPCR Master mix. Primer sequences were delivered by Qiagen (miRCURY LNA miRNA PCR Assay). Detailed information about primers was described by Sikorska et al.^18^. Primers for reference gene U6 were based on the literature (F: GGAACGATACAGAGAAGATTAGC; R: TGGAACGCTTCACGAATTTGCG; Du et al.^38^). Thermal cycling was conducted in LightCycler 480 instrument II (Roche Applied Science, Basel, Switzerland). Analysis was performed in 5 biological replications in each group and two technical repeats for each sample. Threshold cycle (Ct) values were generated using LightCycler 480 software, version 1.5 (Roche Applied Science, Basel, Switzerland), to calculate relative gene expression. Relative analysis of individual miRNA expression was performed using the ddCt method described by Livak and Schmittgen^39^. The ddCt method is a comparative method based on the mathematical model represented by the following formula: R = 2^–ΔΔC^.

**Table 1:** Changes in miRNA expression read from the expression microarray in cecal tonsils and spleen after administration of bioactive substances in ovo on day 12 of egg incubation.

	Fold change	ANOVA p-value	Tissue	Substance	Number of regulated genes^a^
miR-1612	− 2.06	0.04*	Cecal tonsils	Synbiotic—*Lactobacillus salivarius* with GOS	1876
miR-204-5p (211)	− 0.76	0.0001*	Spleen	Prebiotic—inulin	271
	− 0.61	0.001*	Spleen	Synbiotic—*Lactococcus lactis* subsp. *cremoris* with GOS	
miR-1674	− 1.60	0.02*	Cecal tonsils	Synbiotic—*Lactobacillus salivarius* with GOS	348
	− 1.67	0.001*	Cecal tonsils	Synbiotic—*Lactococcus lactis* subsp. *lactis* with inulin	
	− 1.48	0.001*	Spleen	Syniotic—*Lactococcus lactis* subsp. *lactis* with inulin	
miR-1652	1.67	0.008*	Spleen	Synbiotic- *Lactobacillus salivarius* with GOS	734
miR-1598	− 0.64	0.002*	Spleen	Synbiotic—*Lactococcus lactis* subsp. *cremoris* with GOS	464
miR-1996 (199b)	− 0.74	0.001*	Cecal tonsils	Synbiotic—*Lactobacillus salivarius* with GOS	467

^a^Quantitative analysis of genes regulated by miRNA data using the TargetScan software (targetscan.org); *P < 0.05, *GOS* galactooligosaccharides.

### Statistical analysis

The relative expression level for individual miRs was compared using the Kruskal–Wallis test. A comparison of rank sums was performed on the dCt values (Ct values for targets miR normalized by the Ct value for the U6-internal control). The dCt values were separately compared within genotypes (GP/Ross 308) and treatment (CON/PRO/PRE/SYN) for the spleen and cecal tonsils. Statistical differences between the compared groups (*p* < 0.05) were determined using the post hoc test of multiple comparisons of Dunn's rank means. Statistical analyzes were performed using GraphPad Prism 9 for Windows (GraphPad Software, San Diego, California USA).

### Functional analysis

The miRNAs which showed significant differences in qPCR results were selected for functional analysis. Target genes of these miRNAs were determined using the miRDB database (http://mirdb.org/), and genes with a target score greater than 80 were selected for higher certainty of the prediction. Gene Ontology (Biological processes (BP), Molecular Functions (MF), Cellular Components (CC), and Immune System Processes (IP) updated on 05.06.2022) enrichment analysis of the selected genes was performed using ClueGO (v2.5.7) plug-in within Cytoscape (v3.9.1)^40^ using *Gallus gallus* as the reference genome. Over-represented gene ontologies (GOs) were identified using a right-sided hypergeometric test with the Benjamin-Hochberg multiple testing correction method. Mid p values ≤ 0.05 were considered as significantly enriched. Significant GOs were clustered based on the Kappa statistics (Kappa score threshold is 0.4) and medium network specificity (GO tree interval; minimum = 3 and maximum = 8). Functions of individual genes were identified using GeneCards—the human gene database^41^.

### Ethics declarations

The experiment was approved by the Local Ethics Committee for Animal Experiments (Bydgoszcz University of Science and Technology, Poland) (study approval reference number 16/2014). All methods were carried out in accordance with relevant guidelines and regulations. All methods are reported in accordance with ARRIVE guidelines (https://arriveguidelines.org) for the reporting of animal experiments.

## Data Availability

All data are available from the corresponding author (Dunislawska Aleksandra) of the manuscript.

## Abbreviations

GO: Gene ontology
miRNA: Micro RNA
GP: Green-legged Partridgelike
CON: Control
PRO: Probiotic
PRE: Prebiotic
GOS: Galactooligosaccharides
SYN: Synbiotic
dCt: Delta Ct values
CT: Cecal tonsils

## References

[1] Shang Y, Kumar S, Oakley B, Kim WK (2018). Chicken gut microbiota: Importance and detection technology. Front. Vet. Sci..

[2] Diaz Carrasco JM, Casanova NA, Fernández Miyakawa ME (2019). Microbiota, gut health and chicken productivity: What is the connection?. Microorganisms..

[3] Rychlik I (2020). Composition and function of chicken gut microbiota. Animals.

[4] Rubio LA (2019). Possibilities of early life programming in broiler chickens via intestinal microbiota modulation. Poult. Sci..

[5] Siwek M, Slawinska A, Stadnicka K, Bogucka J, Dunislawska A, Bednarczyk M (2018). Prebiotics and synbiotics: In ovo delivery for improved lifespan condition in chicken. BMC Vet. Res..

[6] Dunislawska A, Herosimczyk A, Ozgo M, Lepczynski A, Ciechanowicz AK, Bednarczyk M (2021). Proteome changes upon in ovo stimulation with Lactobacillus synbiotic in chicken liver. Poult. Sci..

[7] Dunislawska A, Herosimczyk A, Lepczynski A, Slama P, Slawinska A, Bednarczyk M (2021). Molecular response in intestinal and immune tissues to in ovo administration of inulin and the combination of inulin and *Lactobacillus lactis* Subsp. cremoris. Front. Vet. Sci..

[8] Bogusławska-Tryk M, Ziółkowska E, Sławińska A, Siwek M, Bogucka J (2021). Modulation of intestinal histology by probiotics, prebiotics and synbiotics delivered in ovo in distinct chicken genotypes. Animals.

[9] Dunislawska A, Slawinska A, Stadnicka K, Bednarczyk M, Gulewicz P, Jozefiak D (2017). Synbiotics for broiler chickens: In vitro design and evaluation of the influence on host and selected microbiota populations following in ovo delivery. PLoS ONE.

[10] Slawinska A, Plowiec A, Siwek M, Jaroszewski M, Bednarczyk M (2016). Long-term transcriptomic effects of prebiotics and synbiotics delivered in ovo in broiler chickens. PLoS ONE.

[11] Riscuta G, Xi D, Pierre-Victor D, Starke-Reed P, Khalsa J, Duffy L, Dumitrescu RG, Verma M (2018). Diet, microbiome, and epigenetics in the era of precision medicine. Cancer Epigenetics for Precision Medicine.

[12] Woo V, Alenghat T (2017). Host–microbiota interactions: Epigenomic regulation. Curr. Opin. Immunol..

[13] Woo V, Alenghat T (2022). Epigenetic regulation by gut microbiota. Gut Microb..

[14] Dunislawska A, Slawinska A, Siwek M, Bednarczyk M (2021). Epigenetic changes in poultry due to reprogramming of the gut microbiota. Anim. Front..

[15] Zhang L, Lu Q, Chang C, Chang C, Lu Q (2020). Epigenetics in health and disease. Epigenetics in Allergy and Autoimmunity.

[16] Dunislawska A, Slawinska A, Siwek M (2020). Hepatic DNA methylation in response to early stimulation of microbiota with lactobacillus synbiotics in broiler chickens. Genes.

[17] Yao Q, Chen Y, Zhou X (2019). The roles of microRNAs in epigenetic regulation. Curr. Opin. Chem. Biol..

[18] Sikorska M, Siwek M, Slawinska A, Dunislawska A (2021). miRNA profiling in the chicken liver under the influence of early microbiota stimulation with probiotic, prebiotic, and synbiotic. Genes.

[19] Dunislawska A, Slawinska A, Gryzinska M, Siwek M (2021). Interaction between early in ovo stimulation of the gut microbiota and chicken host: Splenic changes in gene expression and methylation. J. Anim. Sci. Biotechnol..

[20] Tavaniello S, Slawinska A, Prioriello D, Petrecca V, Bertocchi M, Zampiga M, Salvatori G, Maiorano G (2022). Effect of galactooligosaccharides delivered in ovo on meat quality traits of broiler chickens exposed to heat stress. Poult. Sci..

[21] Madej JP, Stefaniak T, Bednarczyk M (2015). Effect of in ovo-delivered prebiotics and synbiotics on lymphoid-organs' morphology in chickens. Poult. Sci..

[22] Villaluenga CM, Wardeńska M, Pilarski R, Bednarczyk M, Gulewicz K (2004). Utilization of the chicken embryo model for assessment of biological activity of different oligosaccharides. Folia Biol..

[23] Slawinska A, Dunislawska A, Plowiec A, Radomska M, Lachmanska J, Siwek M, Tavaniello S, Maiorano G (2019). Modulation of microbial communities and mucosal gene expression in chicken intestines after galactooligosaccharides delivery in ovo. PLoS ONE.

[24] Dunislawska A, Slawinska A, Bednarczyk M, Siwek M (2019). Transcriptome modulation by in ovo delivered Lactobacillus synbiotics in a range of chicken tissues. Gene.

[25] Pietrzak E, Dunislawska A, Siwek M, Zampiga M, Sirri F, Meluzzi A, Tavaniello S, Maiorano G, Slawinska A (2020). Splenic gene expression signatures in slow-growing chickens stimulated in ovo with galactooligosaccharides and challenged with heat. Animals.

[26] Dunislawska A, Siwek M, Slawinska A, Lepczynski A, Herosimczyk A, Kolodziejski PA, Bednarczyk M (2020). Metabolic gene expression in the muscle and blood parameters of broiler chickens stimulated in ovo with synbiotics. Animals.

[27] Lee JY, Jeong W, Lim W, Lim CH, Bae SM, Kim J, Bazer FW, Song G (2013). Hypermethylation and post-transcriptional regulation of DNA methyltransferases in the ovarian carcinomas of the laying hen. PLoS ONE.

[28] Madej JP, Skonieczna J, Siwek M, Kowalczyk A, Łukaszewicz E, Slawinska A (2020). Genotype-dependent development of cellular and humoral immunity in the spleen and cecal tonsils of chickens stimulated in ovo with bioactive compounds. Poult. Sci..

[29] Siwek M, Wragg D, Sławińska A, Malek M, Hanotte O, Mwacharo JM (2013). Insights into the genetic history of Green-legged Partridgelike fowl: mtDNA and genome-wide SNP analysis. Anim. Genet..

[30] Koenen ME, Boonstra-Blom AG, Jeurissen SHM (2002). Immunological differences between layer- and broiler-type chickens. Vet. Immunol. Immunopathol..

[31] Van Der Most PJ, De Jong B, Parmentier HK, Verhulst S (2011). Trade-off between growth and immune function: A meta-analysis of selection experiments. Funct Ecol..

[32] Teng Y, Ren Y, Sayed M, Hu X, Lei C, Kumar A, Hutchins E, Mu J, Deng Z, Luo C, Sundaram K, Sriwastva MK, Zhang L, Hsieh M, Reiman R, Haribabu B, Yan J, Jala VR, Miller DM, Zhang HG (2018). Plant-derived exosomal microRNAs shape the gut microbiota. Cell Host Microb..

[33] Brisbin JT, Gong J, Parvizi P, Sharif S (2010). Effects of lactobacilli on cytokine expression by chicken spleen and cecal tonsil cells. Clin. Vaccine Immunol. CVI..

[34] Hasan N, Yang H (2019). Factors affecting the composition of the gut microbiota, and its modulation. PeerJ.

[35] Liu S, Da Cunha AP, Rezende RM, Cialic R, Wei Z, Bry L, Comstock LE, Gandhi R, Weiner HL (2016). The host shapes the gut microbiota via fecal microRNA. Cell Host Microb..

[36] Rodríguez-Nogales A, Algieri F, Garrido-Mesa J, Vezza T, Utrilla MP, Chueca N, García F, Rodríguez-Cabezas ME, Gálvez J (2018). Intestinal anti-inflammatory effect of the probiotic *Saccharomyces boulardii* in DSS-induced colitis in mice: Impact on microRNAs expression and gut microbiota composition. J. Nutr. Biochem..

[37] Gleeson PA (2014). The role of endosomes in innate and adaptive immunity. Semin. Cell Dev. Biol..

[38] Du J, Gao S, Tian Z, Xing S, Huang D, Zhang G (2017). MicroRNA expression profiling of primary sheep testicular cells in response to bluetongue virus infection. Infect. Genet. Evol..

[39] Livak KJ, Schmittgen TD (2001). Analysis of relative gene expression data using real-time quantitative PCR and the 2−ΔΔCT method. Methods.

[40] Bindea G, Mlecnik B, Hackl H, Charoentong P, Tosolini M, Kirilovsky A (2009). ClueGO: A Cytoscape plug-in to decipher functionally grouped gene ontology and pathway annotation networks. Bioinform. Oxf. Engl..

[41] Safran M, Rosen N, Twik M, BarShir R, Stein TI, Dahary D, Abugessaisa I, Kasukawa T (2021). The gene cards suite. Practical Guide to Life Science Databases.

